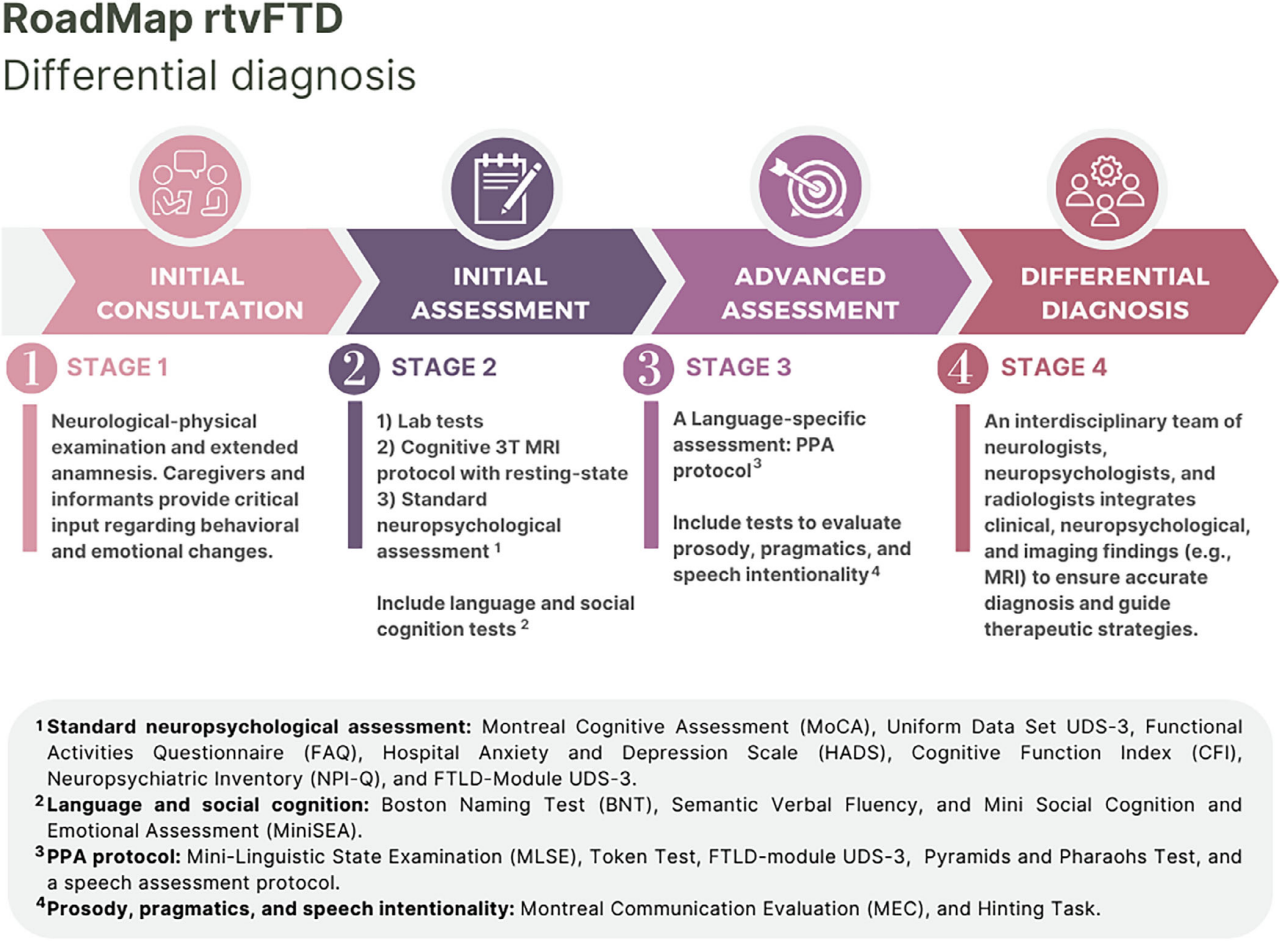# A RoadMap for Neuropsychological Assessment of the Right Temporal Variant of Frontotemporal Dementia (rtvFTD): Case Studies and Practical Applications

**DOI:** 10.1002/alz70857_105781

**Published:** 2025-12-25

**Authors:** Loana De Los Santos, Florentina Morello Garcia, Carolina Agata Ardohain Cristalli, Maria Eugenia Tabernero, Maria Florencia Clarens, Luc¡a Crivelli, Nahuel Magrath Guimet

**Affiliations:** ^1^ Fleni, Buenos Aires, Buenos Aires, Argentina; ^2^ Institute of Neurosciences (INEU), Fleni‐CONICET, Buenos Aires, Buenos Aires, Argentina; ^3^ Fleni, Buenos Aires, Argentina; ^4^ GBHI, San Francisco, CA, USA

## Abstract

**Background:**

The right temporal variant of frontotemporal dementia (rtvFTD) is a neurodegenerative condition characterized by progressive atrophy of the right anterior temporal lobe (rATL), significantly impairing semantic‐pragmatic comprehension and social cognition. In Latin America, although magnetic resonance imaging (MRI) and computed tomography (CT) are widely available, there is still a need for neuropsychological tools to assess cognitive and social changes in rtvFTD. Currently, this condition remains a subject of debate due to diagnostic challenges stemming from a lack of consensus in terminology and variability in assessment tools (Ulugut et al., 2024; Younes et al., 2022). The aim of this study is to propose neuropsychological tools to characterize both the profile and cognitive changes of rtvFTD and present a structured roadmap to help differentiate rtvFTD from other dementias. Additionally, this roadmap contributes to the design of personalized therapeutic interventions.

**Method:**

Two clinical cases diagnosed with rtvFTD at FLENI (Buenos Aires, Argentina) were studied. Both patients underwent standard neuropsychological evaluations focused on semantic‐pragmatic language and social cognition, using locally adapted tests for naming, semantic verbal fluency, semantic association, prosody, pragmatics, and speech intentionality. Findings were correlated with MRI scans to validate the proposed roadmap.

**Result:**

The patients exhibited severe deficits in naming, semantic verbal fluency, semantic‐pragmatic impairments, and alterations in emotional prosody, theory of mind, and facial emotion recognition. Executive attentional systems, visuospatial abilities, and memory remained preserved. These findings aligned with patterns of atrophy and hypometabolism observed in the rATL and were consistent with current literature on the neuropsychological and clinical profiles of the rtvFTD. Figure 1 shows the proposed neuropsychological assessment approach, using a regionally adapted cognitive battery designed to capture rtvFTD symptoms in Spanish‐speaking populations and to guide differentiation from other dementia variants.

**Conclusion:**

This roadmap provides a practical guide that includes neuropsychological tests for the assessment of rtvFTD, particularly in Spanish‐speaking countries. By integrating evaluations targeting semantic‐pragmatic language and social cognition, the roadmap allows for precise differentiation of rtvFTD from other frontotemporal dementia variants. Furthermore, it contributes to the development of personalized therapeutic interventions, aiming to improve patient quality of life and support clinical practices in Spanish‐speaking regions.